# *Notes from the Field*: Xylazine Detection and Involvement in Drug Overdose Deaths — United States, 2019

**DOI:** 10.15585/mmwr.mm7037a4

**Published:** 2021-09-17

**Authors:** Mbabazi Kariisa, Priyam Patel, Herschel Smith, Jessica Bitting

**Affiliations:** ^1^Division of Overdose Prevention, National Center for Injury Prevention and Control, CDC; ^2^Oak Ridge Institute for Science and Education, Oak Ridge, Tennessee; ^3^National Network of Public Health Institutes, New Orleans, Louisiana.

Xylazine is a drug used in veterinary medicine as an animal sedative with muscle relaxant and analgesic properties (*1*). It is not approved by the Food and Drug Administration for use in humans, in whom it acts as a central nervous system depressant and can cause respiratory depression, slowed heart rate, and hypotension (*2*). When used as a toxic adulterant in illicitly produced opioids such as fentanyl or heroin (*3*), xylazine might potentiate sedation and respiratory depression, increasing the risk for fatal overdose. In addition, because xylazine is not an opioid, it does not respond to opioid reversal agents such as naloxone; therefore, if illicit opioid products containing xylazine are used, naloxone might be less effective in fully reversing an overdose. Several states have reported increases in xylazine-involved overdose deaths; however, the prevalence of xylazine involvement in drug overdose deaths (overdose deaths) has not been extensively studied, particularly in the United States (*4*). To better understand the impact of xylazine adulteration on the evolving drug overdose epidemic in the United States, CDC analyzed unintentional and undetermined intent overdose death data from the State Unintentional Drug Overdose Reporting System (SUDORS) in 38 states and the District of Columbia (DC).*^,^^†^

A SUDORS case was defined as xylazine-positive if xylazine was detected on postmortem toxicology or if xylazine was listed on the death certificate as a contributing cause of death by the medical examiner or coroner based on postmortem toxicology detection, evidence of drug use at the scene, or witness reports of drug use. SUDORS cases in which xylazine is listed on the death certificate as a contributing cause of death by the medical examiner or coroner were defined as xylazine-involved. Thus, a xylazine-involved case would also be considered to be xylazine-positive by definition; however, a xylazine-positive case would not always mean that xylazine contributed to the death (i.e., xylazine-involved). Using data from 38 states and DC, CDC examined xylazine-positive and xylazine-involved overdose deaths that occurred during 2019. In addition, detailed narrative text for each case was reviewed for information about xylazine use or presence among drug products or paraphernalia found at the scene.

Among 45,676 overdose deaths reported to SUDORS during January–December 2019, xylazine-positive (826; 1.8%), and xylazine-involved (531; 1.2%) deaths were identified in 25 and 23 states, respectively. Xylazine was listed as a cause of death in 64.3% of deaths in which it was detected. The majority of xylazine-involved deaths were among males (73.1%), non-Hispanic White persons (75.4%), and from states in the Northeast Census region (67.0%).^§^ Among all xylazine-involved deaths, one or more other drugs, particularly illicit drugs, were also listed as a cause of death, and 98.7% of xylazine-positive deaths and 99.1% of xylazine-involved deaths had fentanyl (including analogs) listed as a cause of death. Cocaine and heroin were listed as a cause of death in 32.1% and 26.0% of xylazine-positive deaths respectively and in 29.6% and 28.4% of xylazine-involved deaths respectively (Table).

**TABLE:** Characteristics of drug overdose decedents with xylazine detected on postmortem toxicology (xylazine-positive) or listed as a cause of death (xylazine-involved) — State Unintentional Drug Overdose Reporting System, 38 states and the District of Columbia,* 2019

Characteristic	Classification of deaths, no. (%)	
	Xylazine-positive^†^ (n = 826)	Xylazine-involved^§^ (n = 531)
**Sex**		
Male	602 (72.9)	388 (73.1)
Female	224 (27.1)	143 (26.9)
**Race** ^¶^		
White, non-Hispanic	604 (74.8)	396 (75.4)
Black, non-Hispanic	106 (13.1)	68 (13.0)
Hispanic	90 (11.1)	—**
Other	11 (1.4)	—**
**Age group, yrs**		
15–24	60 (7.3)	41 (7.7)
25–34	265 (32.1)	181 (34.1)
35–44	227 (27.5)	138 (26.1)
45–54	147 (17.8)	91 (17.1)
55–64	109 (13.2)	—**
≥65	18 (2.2)	—**
**U.S. Census region** ^††^		
Northeast	568 (68.8)	356 (67.0)
Midwest	144 (17.4)	91 (17.1)
South	104 (12.6)	—**
West	10 (1.2)	—**
**Co-occurring drugs listed as a cause of death** ^§§^ **^,^** ^¶¶^		
Any fentanyl (including analogs)	815 (98.7)	526 (99.1)
Heroin***	215 (26.0)	151 (28.4)
Benzodiazepines	141 (17.1)	105 (19.8)
Prescription opioids^†††^	94 (11.4)	71 (13.4)
Cocaine	265 (32.1)	157 (29.6)
Alcohol	98 (11.9)	67 (12.6)
Methamphetamine	102 (12.4)	62 (11.7)

**Abbreviation**: SUDORS = State Unintentional Drug Overdose Reporting System.

* Thirty-nine jurisdictions reported data during January–December 2019, including 29 that reported deaths that occurred during the entire period: Alaska, California, Connecticut, District of Columbia, Delaware, Georgia, Illinois, Indiana, Kentucky, Maine, Massachusetts, Minnesota, Missouri, Nevada, New Hampshire, New Jersey, New Mexico, North Carolina, Ohio, Oklahoma, Pennsylvania, Rhode Island, Tennessee, Utah, Vermont, Virginia, Washington, West Virginia, and Wisconsin. Four additional states only reported deaths that occurred during January–June 2019: Florida, Louisiana, Maryland, and Michigan. Six states only reported deaths that occurred during July–December 2019: Arizona, Colorado, Kansas, Montana, Oregon, and South Dakota. Thirty-one jurisdictions abstracted data on all drug overdose deaths within the jurisdiction and seven states (California, Florida, Illinois, Indiana, Louisiana, Missouri, and Washington) abstracted data on deaths within a subset of counties accounting for at least 75% of that state’s overdose deaths in 2017, or at least 1,500 overdose deaths. Data were current as of December 10, 2020.

^†^ A SUDORS case was defined as xylazine-positive if xylazine was detected on postmortem toxicology or if xylazine was listed as a contributing cause of death by the medical examiner or coroner on the death certificate. The medical examiner or coroner determines if xylazine was involved or contributed to the death based on postmortem toxicology detection, evidence of drug use at the scene or witness reports of drug use.

^§^ SUDORS cases that had xylazine listed as a contributing cause of death by the medical examiner or coroner were defined as xylazine-involved. Thus, a xylazine-involved case would also be considered to be xylazine-positive by definition; however, a xylazine-positive case would not always mean that xylazine contributed to the death (i.e., xylazine-involved).

^¶^ Race and ethnicity data were missing for 15 xylazine-positive decedents. Race and ethnicity data were missing for six xylazine-involved decedents.

** Cells with ≤9 deaths are not reported. Some cells are not reported to prevent calculation of another suppressed cell.

^††^ *Northeast*: Connecticut, Maine, Massachusetts, New Hampshire, New Jersey, New York, Pennsylvania, Rhode Island, and Vermont; *Midwest*: Illinois, Indiana, Iowa, Kansas, Michigan, Minnesota, Missouri, Nebraska, North Dakota, Ohio, South Dakota, and Wisconsin; *South*: Alabama, Arkansas, Delaware, District of Columbia, Florida, Georgia, Kentucky, Louisiana, Maryland, Mississippi, North Carolina, Oklahoma, South Carolina, Tennessee, Texas, Virginia, and West Virginia; *West*: Alaska, Arizona, California, Colorado, Hawaii, Idaho, Montana, Nevada, New Mexico, Oregon, Utah, Washington, and Wyoming.

^§§^ Identified as a cause of death by a medical examiner or coroner.

^¶¶^ Multiple drugs could be listed as a cause of death; therefore, drugs are not mutually exclusive.

*** Drugs coded as heroin were heroin and 6-monoacetylmorphine. In addition, morphine was coded as heroin if detected along with 6-acetylmorphine or if scene, toxicology, or witness evidence indicated presence of heroin impurities or other illicit drugs, injection, illicit drug use, or a history of heroin use.

^†††^ Drugs coded as prescription opioids were alfentanil, buprenorphine, codeine, dextrorphan, hydrocodone, hydromorphone, levorphanol, loperamide, meperidine, methadone, morphine, noscapine, oxycodone, oxymorphone, pentazocine, prescription fentanyl, propoxyphene, remifentanil, sufentanil, tapentadol, and tramadol. Also included as prescription opioids were brand names (e.g., Opana) and metabolites (e.g., nortramadol) of these drugs and combinations of these drugs and nonopioids (e.g., acetaminophen-oxycodone). Morphine was included as prescription only if scene or witness evidence did not indicate likely heroin use and if 6-acetylmorphine was not also detected. Fentanyl was coded as a prescription opioid based on scene, toxicology, or witness evidence.

The findings in this report are subject to at least one limitation. Estimates of xylazine detection in overdose deaths might be underestimated. Data reviews revealed instances where xylazine presence was noted at the scene of the overdose but not detected on postmortem toxicology. Routine postmortem toxicology panels might not have included tests for xylazine, and current testing protocols for xylazine are not standard, which could result in missed detection (*5*).

During 2019, fewer than 2% of SUDORS overdose deaths from 38 states and DC were xylazine-positive. Xylazine contributed to death in approximately one half of deaths in which it was detected and was primarily co-involved with fentanyl. The detection of xylazine and its involvement in overdose deaths in multiple jurisdictions is concerning and warrants continued surveillance to inform overdose response and prevention efforts. Naloxone administration might not be as effective at fully reversing overdose-related signs and symptoms when xylazine and highly potent opioids such as fentanyl are present, although naloxone should always be administered. No pharmaceutical antidote is specific to xylazine, and immediate supportive care, especially respiratory and cardiovascular support, is critical in the event of an overdose when the presence of xylazine is suspected. Implementing routine standardized postmortem toxicology testing protocols for xylazine could help better elucidate the role of xylazine in drug overdose deaths. 

## Footnotes

* SUDORS captures data on fatal unintentional and undetermined intent overdoses. For all captured overdose deaths, SUDORS records all drugs detected by postmortem toxicology, even those not ruled by a medical examiner or coroner to have contributed to the death. A drug was recorded as contributing to death when the death certificate or medical examiner or coroner report listed the drug as a contributing factor.

† Thirty-eight states and DC reported data during January 2019–December 2019. Twenty-nine jurisdictions reported deaths that occurred during the entire period: Alaska, California, Connecticut, District of Columbia, Delaware, Georgia, Illinois, Indiana, Kentucky, Maine, Massachusetts, Minnesota, Missouri, Nevada, New Hampshire, New Jersey, New Mexico, North Carolina, Ohio, Oklahoma, Pennsylvania, Rhode Island, Tennessee, Utah, Vermont, Virginia, Washington, West Virginia, and Wisconsin. Four additional states only reported deaths that occurred during January–June 2019: Florida, Louisiana, Maryland, and Michigan. Six states only reported deaths that occurred during July–December 2019: Arizona, Colorado, Kansas, Montana, Oregon, and South Dakota. Thirty-one jurisdictions abstracted data on all drug overdose deaths within the jurisdiction and seven states (California, Florida, Illinois, Indiana, Louisiana, Missouri, and Washington) abstracted data on deaths within a subset of counties accounting for at least 75% of that state’s overdose deaths in 2017, or at least 1,500 overdose deaths. Data were current as of December 10, 2020.

§ Connecticut, Maine, Massachusetts, New Hampshire, New Jersey, New York, Pennsylvania, Rhode Island, and Vermont.
